# Nanomaterials in Cementitious Composites: An Update

**DOI:** 10.3390/molecules26051430

**Published:** 2021-03-06

**Authors:** Zoi S. Metaxa, Athanasia K. Tolkou, Stefania Efstathiou, Abbas Rahdar, Evangelos P. Favvas, Athanasios C. Mitropoulos, George Z. Kyzas

**Affiliations:** 1 Department of Chemistry, International Hellenic University, GR-654 04 Kavala, Greece; stefaniaefs1@gmail.com (S.E.); amitrop@chem.ihu.gr (A.C.M.); 2 Laboratory of Chemical and Environmental Technology, Department of Chemistry, Aristotle University of Thessaloniki, GR-541 24 Thessaloniki, Greece; tolkatha@chem.auth.gr; 3 Department of Physics, Faculty of Science, University of Zabol, Zabol 98613-35856, Iran; a.rahdar@uoz.ac.ir; 4 Institute of Nanoscience and Nanotechnology, NCSR “Demokritos”, Aghia Paraskevi, GR-153 41 Athens, Greece; e.favvas@inn.demokritos.gr

**Keywords:** cementitious nano-composites, nanomaterials, mechanical properties

## Abstract

This review is an update about the addition of nanomaterials in cementitious composites in order to improve their performance. The most common used nanomaterials for cementitious materials are carbon nanotubes, nanocellulose, nanographene, graphene oxide, nanosilica and nanoTiO_2_. All these nanomaterials can improve the physical, mechanical, thermal and electrical properties of cementitious composites, for example increase their compressive and tensile strength, accelerate hydration, decrease porosity and enhance fire resistance. Cement based materials have a very complex nanostructure consisting of hydration products, crystals, unhydrated cement particles and nanoporosity where traditional reinforcement, which is at the macro and micro scale, is not effective. Nanomaterials can reinforce the nanoscale, which wasn’t possible heretofore, enhancing the performance of the cementitious matrix.

## 1. Introduction

Cement is the second single most widely used material after water. In 2014 the global cement production had already exceeded 4.18 billion tones [1]. Cementitious composites are preferred due to their high compressive strength, low-cost preparation, simple production process and convenience of use [2]. However, these composites have many disadvantages such as low tensile capacity, poor deformation performance and high cracking tendency which affect the long-term durability of structures. Furthermore, in harsh environments they typically suffer from physical, chemical and biological damage leading to degradation and service life shortening. Due to its porous structure [3], in most cases the attack and deterioration initiates from the surface of the material.

Several studies have shown that the interfacial transition zone (ITZ) in concrete, i.e., the zone surrounding the aggregates, is the weakest part in concrete having relatively large pores and high porosity, higher than the porosity of bulk cement paste [4]. It is obvious that porosity in cementitious materials play a major role because provides the primary transport route of substances in and out of cement-based materials, so the size and the structural features of pores affect the mechanical properties, the fluid diffusion characteristics and finally the durability of the material [5,6]. Moreover, it is well known that concrete demonstrates a very low tensile strength (2–8 MPa) which typically is ten times smaller than its compressive strength [7]. It is important to overcome all these issues in cement-based materials so the durability of structures could be improved. The common method of improvement includes the use of reinforcement in macro and micro scale, like fibers and fillers, but cementitious matrices demonstrate defects/porosity at the nanoscale, where traditional reinforcement is not effective.

It is well known that nanoscale materials demonstrate excellent physical and chemical characteristics presenting improved mechanical, electrical and thermal properties, low density and excellent chemical and thermal stability. A large number of different nanomaterial types exist so cementitious nanocomposites with very different properties can be designed improving the life-cycle of cementitious materials [8]. Nanostructures such as nanofibers, nanotubes and nanoparticles like nano-TiO_2_ and nano-SiO_2_ can be used to reinforce the cementitious matrix developing a new generation of high-performance, and multifunctional cementitious composites that was not possible heretofore.

**Figure 1:**
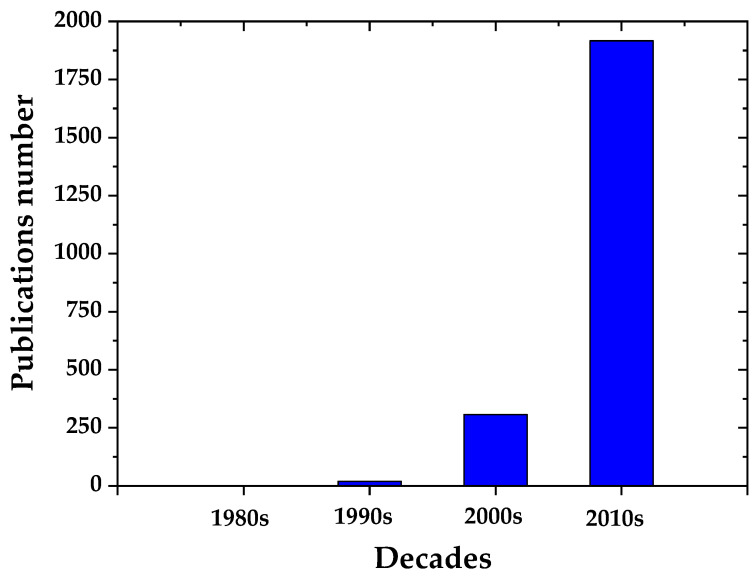
Publications on nanotechnology in cementitious composites per decade.

**Figure 2:**
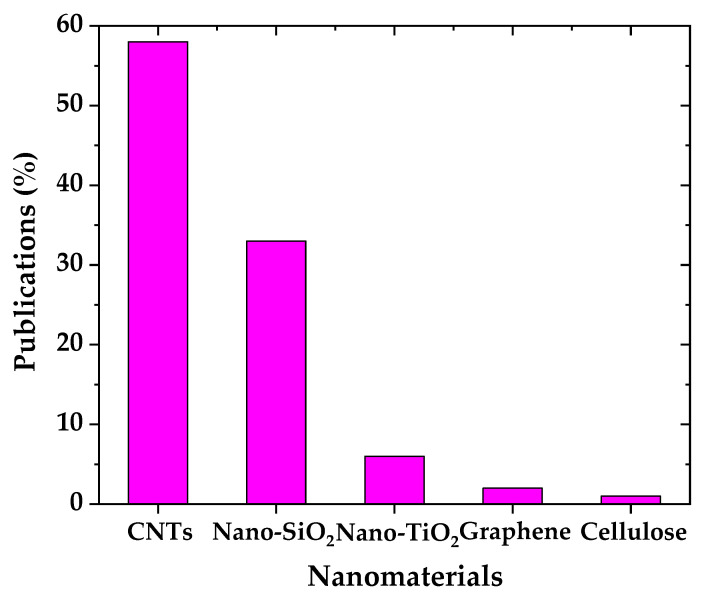
Publications (%) on each nanomaterial with application on the construction sector.

In the past decade, the publications concerning nanotechnology in the construction sector have increased dramatically (Figure 1). Analyzing the data, it was observed that the main focus (~58% of the total publications) has been on the incorporation of carbon nanotubes (CNTs) (Figure 2) followed by nanosilica (~34% of the total publications) and nanotitania (~7% of the total publications). The studies are mainly focusing on the nanomaterials’ effect on the performance of the cementitious matrix without addressing their possible impact on human health and the environment. Therefore, it is mandatory to address the main nanomaterials’ drawbacks. Unfortunately, there are conflicting results in the literature regarding the pathologic effects of nanomaterials. As a result, their possible interaction with our biological system is still unknown. Additionally, their increased cost, compared to the same materials at a larger scale, is a considerable drawback, however, in most cases their optimum concentration is quite low making their cost comparable or even lower than conventional materials. Moreover, the fact that in order to take advantage of their excellent properties they need to be well dispersed into the matrix adds an additional step on the development of the composites that could be an issue at large scale applications. Finally, the nanocomposites developed should be evaluated in relation to sustainability and their environmental and economic consequences. A recent review, includes additional information on some of the recent concrete nanocomposites, analyzing them on the spectrum of ecological sustainability, and economic benefits [8].

The present review article provides information on the impact of all the different types of nanomaterials typically used for reinforcing cement-based materials such as carbon nanotubes, nanosilica and nano-TiO_2_, nanographene, graphene oxide and nanocellulose. Important information on the structure, synthesis, dispersion methods and key performance characteristics of nanocomposites produced using the different nanomaterials studied is provided. Moreover, information on the effect that different nanomaterials have on the mechanical properties (compressive and flexural strength, fracture toughness and elastic modulus), hydration, porosity, smart properties, and other properties (fire resistance, freeze-thaw and electromagnetic adsorption) of the cementitious matrix is presented.

## 2. Nanomaterials Typically Used in Cement-Based Materials

### 2.1. Carbon Nanotubes

Carbon nanotubes (CNTs) consist of rolled graphite nanosheets and are typically divided in two general types. The single-walled nanotubes (SWCNTs), which were developed in 1993, and have only one wall forming a tube, and multi-walled nanotubes (MWCNTs), having multiple tubes which can slide against each other. The diameter of CNTs is between 1 and 100 nm and the surface area is usually in the range of 100–700 m^2^/g [9]. The diameter of SWCNTs range from 0.4 to 3 nm, while their length ranges from 1 to 50 μm. The diameter of MWCNTs range from 1.4 to 100 nm and their length range from 0.1 to 100 μm. Their aspect ratio is typically around 1000 [10]. The density depends on the diameter, number of walls in MWCNTs and the length. SWCNTs have high cost, so MWCNTs are preferable in cement-based materials.

#### 2.1.1. Dispersion

**Figure 3:**
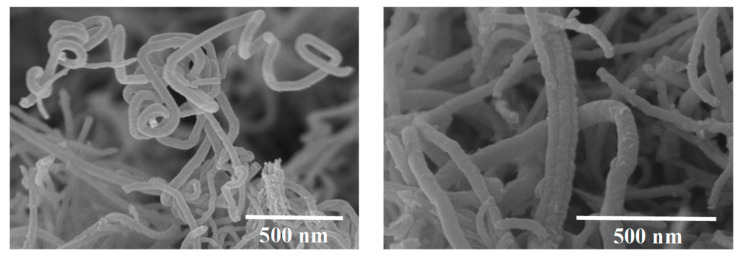
SEM images of MWCNTs forming bundles. Reproduced from Konsta-Gdoutos et al. [18] with permission from Elsevier.

One very important factor when preparing CNTs reinforced cementitious materials is the CNTs distribution inside the matrix. Due to the van der Waals forces CNTs have the tendency to agglomerate and form bundles (Figure 3), when used as received leading to a drop in the mechanical performance. It has been proved that conventional concrete mixers are not able to disperse CNTs into cement paste directly [11]. So usually, the CNTs are dispersed first into water and then are mixed with the cementitious particles. There are physical and chemical methods for CNTs dispersion. Some known techniques are sonication, ball milling, mechanical stirring and surfactants. Ball milling is a powder milling method which is used in order to break the agglomerations of CNTs formed by van der Waals forces and it is suitable when dispersing CNTs in high concentrations [12]. Nevertheless, this method decreases the CNTs’ aspect ratio [13,14]. Mechanical stirring is a shear mixing method, which usually is used with sonication, but is not able to disperse CNTs well in aqueous solutions, therefore it is used as a preliminary treatment for the CNTs suspension [15,16]. Sonication provides the required energy to overcome the van der Waals interactions [17]. Surfactants with long chains can be adsorbed on the CNTs, the hydrophobic part of the surfactant is adsorbed at the sidewalls or the end of the tubes through van der Waals forces [10]. An effective dispersion of CNTs in water can be achieved by applying ultrasonic energy and in combination with the use of a surfactant [18]. Another dispersion technique has been suggested using Pluronic F-127 as a novel dispersing agent. This method increases stiffness, fracture energy and ductility [19].

The most common method to estimate the stability and quality of CNT dispersion, is ultraviolet-visible (UV-vis) spectroscopy [20]. Different structures can cause different characteristic peaks, in terms of chirality and diameter. The disadvantage of this method is that it is not able to speculate the shortening effect of sonication on the CNTs [21,22] Scanning electron microscopy (SEM) is the most commonly used method for assessment of dispersion of CNTs in hardened cement. This method is able to intuitively reflect the distribution of CNTs and the failure mode of the bond between CNTs and the cementitious hydration products [18,23,24,25].

#### 2.1.2. Cement Hydration

According to several studies, the addition of CNTs to cement matrix can accelerate the hydration of cement at approximately 78%. This acceleration has many advantages such as earlier finishing of surfaces, reduction of the hydraulic pressure on forms, sooner removing forms, decrease of curing time and compensation for the reaction of low temperature on strength development [26,27,28,29,30].

#### 2.1.3. Porosity, Water Absorption, Permeability and Microstructure

The addition of CNTs in the matrix decreases its porosity, water absorption and permeability, because CNTs can fill in the pores between the cement hydration products [31,32,33,34]. This is a great advantage because porosity, water absorption and permeability are the main factors that affect durability and service life of the material. Microstructure analysis by SEM and MIP tests have indicated that MWCNTs with diameter of 10–20 nm demonstrate the best effect on optimizing pore structure [35]. A recent study combining nanoindentation with elemental mapping and X-ray scanning microtomography shows that higher density hydrated phases are forming at a CNTs concentration of 0.5 wt% [36]. In general, porosity increases with increasing diameter of MWCNTs. Smaller diameter of MWCNTs effectively improve the pore size distribution and reduce porosity in cement-based materials resulting to a denser microstructure [35,37].

#### 2.1.4. Mechanical Properties

CNTs are well known to improve the mechanical performance of the cementitious matrix. According to a recent review [38], the incorporation of CNTs in cement paste can result to a maximum improvement in the compressive and flexural strength of 83.33% [39] and 30% [34], respectively. In case of a mortar matrix, the percentages are dropping reaching a maximum of ~35% for the compressive strength [40] and 28.04% for the flexural strength [41]. When a concrete matrix is used, the maximum improvements achieved are similar (38.62% and 38.63%, respectively) [42]. In all the above studies, several optimum CNT percentages were used ranging from 0.02 up to 0.5 wt%.

The concentration of CNTs is one of the determining factors of the mechanical performance of CNT/cement nanocomposites. Typically, the compressive and flexural strength of the matrix increases with the CNT addition until a certain concentration is reached [43,44,45]. Once the optimum concentration is exceeded, it starts decreasing. The observed reductions at high CNT concentrations occur because CNTs are forming agglomerations within the matrix. As a result, localized stresses are developed weakening the strength of the nanocomposite.

#### 2.1.5. Electrical Properties

Cement-based materials generally have low electrical conductivity and no self-sensing behavior. CNTs can form an electrically conducting network inside the insulating matrix [46] and enhance its electrical conductivity [47], when they are used at an optimum amount [47,48]. The addition of CNTs into cement paste results to a sharp decrease in the electrical resistance [49,50] and, at the same time provides the matrix with piezoresistive characteristics [49,51,52]. Compared to other carbon nanomaterial types CNTs demonstrate better self-sensing characteristics under cyclic compression [53]. The electrical properties of the CNT nanocomposites can be affected by numerous factors: CNTs content, dispersion level, intensity and frequencies, w/c ratio, moisture content, etc.

#### 2.1.6. Durability

The potential of cementitious CNT nanocomposites to resist degradation caused by the surrounding environment can be understood by studying their durability properties. Several studies have been published, showing the effects of CNTs on autogenous [54,55,56] and drying shrinkage [57,58,59], creep [60], carbonation [61], chloride ion penetration [61,62,63] etc. In all of the studies reported above, a positive impact on the durability performance of the nanocomposite was observed with the CNT addition. Only Dalla et al. [64], in contrast to the results reported by other authors, have reported an increasing trend in the chloride ion penetration possibly because the measurement reflects not only chloride ions, but the total of ions contained to the mixture.

### 2.2. Cellulose

#### 2.2.1. Cellulose Nanocrystals

**Figure 4:**
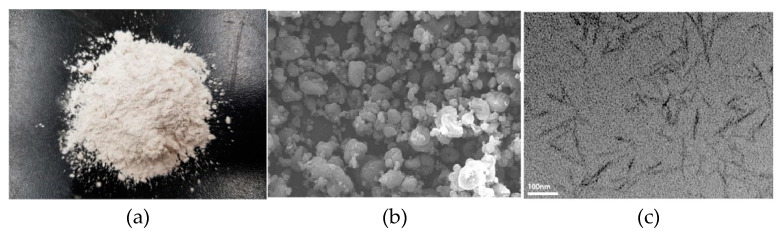
(**a**) Raw CNCs; (**b**) their scanning electron microscopy image; (**c**) transmission electron microscope image of dispersed CNCs. Reproduced from Lee et al. [66] with permission from Elsevier.

Cellulose nanocrystals (CNCs) are rod-like nanoparticles (usually 0.05–0.5 μm in length and 3–5 nm in width) which can be extracted from plants and trees [65]. They are typically produced in powder form. Raw CNCs, their morphology (SEM image) and transmission electron microscope image of dispersed CNCs are shown in Figure 4 [66]. They have some special properties like high elastic modulus and strength, low density, reactive surfaces, which allow functionalization and are immediately water-dispersible without the use of surfactant or modification [65]. More advantages of CNCs are their renewability, sustainability, low toxicity, low cost (evaluated production costs of <10$/1b). Because they are extracted from physical sources like plants and trees, CNCs are biodegradable, carbon neutral and have not environmental and health risk [65,67]. One method to extract the CNCs is through sulfuric acid hydrolysis of Eucalyptus dry-lap cellulose fibers, resulting in a 0.81 wt.% CNC surface-grafted sulfate content [68]. The CNC source and production method strongly affects the properties of the CNC-cementitious nanocomposites [69].

The CNCs in the fresh cement paste are separated in two types: the ‘‘free’’ CNCs (fCNCs) in the water and the ‘‘adsorbed’’ CNCs (aCNCs) on cement surface. Both types of CNCs are in solution, the main difference between them is that the aCNCs are difficult to move, as they stick to the cement particles and the fCNCs can unobstructedly move in the aqueous suspension. The majority of CNCs (>94%) are aCNCs [70].

CNCs can enhance the mechanical performance of cementitious composites. Thanks to their small size could possibly reduce inner fiber spacing, prevent micro-cracking and therefore increase the strength of the matrix [68]. They can also increase the flexural strength of cement paste about 50% [71], increase the compressive strength by 27% [72] and 44% when surface modified [73]. Possibly they can increase the degree of hydration, especially at early age [74], because they could provide a channel for water transporting through the hydration products ring to the unhydrated cement particles [68]. They were also found to improve the frost resistance after 50 freeze-thaw cycles [72]. As expected, CNC were found to have no effect or slightly increase the resistivity of the matrix [75]. At low concentrations (<0.2%), CNC can improve cement rheology reducing yield stress by up to 54% [76]. On the contrary, at much higher amounts (>0.5%), a tenfold increase in the yield strength is observed [76]. The incorporation of CNCs reduces the porosity, improving the microstructure of the matrix [77].

#### 2.2.2. Cellulose Filaments

**Figure 5:**
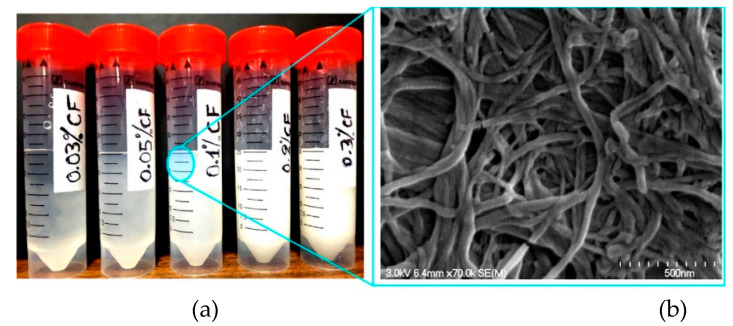
Cellulose filaments (CF): (**a**) CF-water suspensions for the different dosages tested in this study; (**b**) Scanning electron microscope (SEM) image of a suspension with 0.10% CF. Reproduced from Hisseine et al. [79] with permission from Elsevier.

Cellulose filaments (CF) are cellulosic fibrils with diameter in the nanoscale, from 30 to 400 nm, and micrometric length of about 100–2000 μm. They have a high aspect ratio of 100–1000 [78]. Figure 5 illustrates a field emission gun-scanning electron microscopy (FEG-SEM) image of a dried 0.10% CF suspension [79]. CF exhibit an intrinsic hydrophilic nature and a hygroscopic character. The hydrophilicity of CF among with their tendency to create a percolating network of filaments plays a crucial role in their viscosity modifying effect [79]. On the other hand, the hygroscopicity of CF affects the internal curing effect observed with CF [80]. CF materials have been found to influence the flexural strength of cement composites up to 25% [81]. Furthermore, CF have reported to increase the compressive strength up to 16%, the splitting tensile strength up to 34% and the energy absorption up to 96% [79,81].

#### 2.2.3. Cellulose Nanofibers

**Figure 6:**
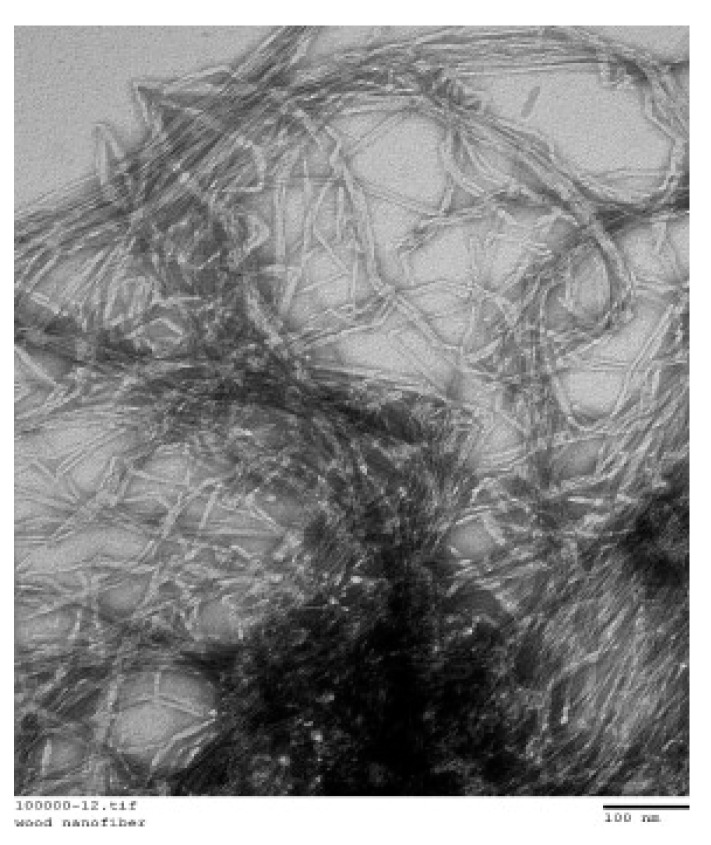
Image of a transmission electron microscope showing the morphology of cellulose nanofibers. Reproduced from Onuaguluchi et al. [85] with permission from Elsevier.

Cellulose nanofibers (CNF) are about 5-500 nm wide and 1–5 μm long with 50–70% crystallinity, possessing electrostatic charge and an extremely high surface area [81,82]. CNFs also have uniform dispersion, high chemical tunability, exceptional hydrophilicity, great colloidal properties and reinforcing potential [81,83,84]. Figure 6 shows a transmission electron microscope image of CNFs in an aqueous solution [85]. All of the studies report that the addition of CNFs improve the degree of hydration [86,87,88,89,90]. CNF can increase both the flexural and compressive strength, possibly because of a higher degree of hydration and densification in the cement paste microstructure, but they lower the workability [91,92,93,94,95]. In particular, the incorporation of CNFs at a low concentration (0.1%) resulted in an increase in flexural strength and energy absorption by 106% and 184%, respectively [85]. Moreover, a 2.7 times increase in the flexural strength of mortars reinforced with algal cellulose nanofibers has been reported [96]. Regarding their effect on durability, the use of carboxyl rich CNF in cement was found to reduce shrinkage and the associated cracking [97]. The sulphate penetration and subsequent dimensional alteration in cementitious systems with CNF has been studied [98]. It was concluded that adding CNF to cementitious systems the sulphate penetration could be decreased. It is possible that nanofibers act as a water reservoir promoting internal curing [98]. CNFs have been reported to be suitable nanoreinforcing material for use in extruded cementitious composites [99].

### 2.3. Graphene

Graphene nanomaterials, that are typically produced from graphite, display a unique atom-thick sp2 bonded 2D structure [100]. Thanks to this structure, graphene has various special properties like ultrahigh tensile strength and elastic modulus, high specific surface area, electrical and optical conductivity [101,102,103].

#### 2.3.1. Graphene Nanoplatelets

**Figure 7:**
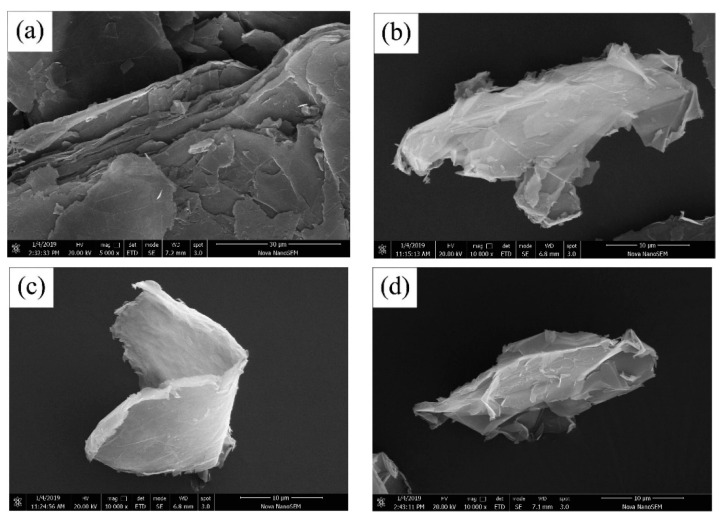
Scanning electron microscopy images showing the morphology of (**a**) as received GNPs; (**b**) GNPs treated with polycarboxylate superplasticizer; (**c**) GNPs treated with naphthalene superplasticizer; (**d**) GNPs treated with melamine superplasticizer. Reproduced from Wang et al. [105] with permission from Elsevier.

Graphene nanoplatelets (GNPs) are nanoparticles formed from graphene stacks. GNPs consist of several layers of graphene sheets with a lateral size (diameter) of a few micrometers and with thickness less than 100 nm [104]. SEM images of GNPs without dispersion (as received) and dispersed with a polycarboxylate superplasticizer (PS), a naphthalene superplasticizer (NS) and a melamine superplasticizer (MS) are shown in Figure 7 [105].

Το aid with their dispersibility in aqueous solutions, GNPs have been treated with polycarboxylate based superplasticizers [106,107,108,109], methylcellulose [110], silica fume [111], polyoxyethylene (40) nonylphenyl ether [112,113], sodium dodecyl benzene sulfonate (SDBS) [114,115] and melamine [116]. The morphology and chemical composition of the polycarboxylate based superplasticizers strongly affects the electrical properties and the self-sensing characteristics of the nanocomposite [106,108]. In most of the cases, the GNP aqueous suspensions were ultra-sonicated to further aid their dispersion [106,107,111]. The reinforcing effect and dispersion of GNPs are both strongly affected by the dispersing agent concentration and the ultrasonic energy application [109]. Raise of GNPs concentration reduces the slump flow and increases the yield stress and plastic viscosity [117,118].

GNP/cementitious nanocomposites demonstrate improved mechanical performance. The use of 0.05% GNPs by weight of cement increased the 28d flexural strength by 16.8% [110] and 25.2% [112]. A low dosage of 0.033% increased the 28d splitting tensile strength by 131.6% [119]. Similarly, a low concentration of 0.06% GNPs increased the compressive and flexural strength by 30.6%, 27.8%, respectively [115]. 2 vol% GNPs increased the compressive strength and elastic modulus of the cementitious matrix by 54% and 50%, respectively [120]. Graphene/cementitious mortars with 0.4 wt% of cement GNPs demonstrated substantial improvements in their fracture behavior with the addition of GNPs. Fracture energies that are up to 1700% higher than the control values are reported [121]. The macroscopic hardness measured with indentation tests was doubled for cement paste reinforced with 1% GNPs [107]. 

GNPs alter the matrix microstructure, refine the pore structure [113,115,122,123], and reduce the porosity [105,107,110,112,115]. A recent in-depth study shows that GNPs depress the meso pores resulting to a denser microstructure [124]. The degree of cement hydration is promoted by GNPs [113,122], especially at an early age [105,110,112,115]. Possibly, the hydrolytic free calcium ions are absorbed by the GNPs leading to the oriented ettringite growth close to the GNPs rather than the cement particles leaving more space on their surface for ion exchange leading to more hydration products production, especially at early age [113]. Nanoindentaion tests have shown a reduction of the porous phase and low-density C-S-H gel and an increase of the high-density C-S-H gel, suggesting the development of a denser microstructure for the graphene-reinforced matrix [125]. The addition of 10% GNPs significantly improves the thermal diffusivity of about 75% at 25 °C and 60% at 400 °C [126]. Similar results were obtained by Piselo et al. [127], GNPs were found the most effective carbon nanomaterial to increase thermal conductivity and diffusivity. A concentration of 2.5% decrease the water penetration depth, chloride diffusion coefficient and chloride migration coefficients by 64%, 70% and 31%, respectively [123]. A more recent study shows that chloride penetration depth and coefficient can be decreased by ~37% and ~42%, respectively with the introduction of GNPs of as little as 0.02% [113]. Rapid chloride penetration tests suggest that GNPs addition decelerate the chloride ions migration [128]. Similar response was obtained by Tong et al. [125], the chemical attack induced by an acidic solution was shown down at the GNP nanocomposites. A 15% reduction in the diffused solar reflectance, uniform in the overall spectrum, has been reported [127]. A slight increase in density (+5.4%) was observed with 0.01wt% GNPs [129].

Increasing the GNPs amount can lower the electric resistivity of the cementitious matrix [118,130,131]. The electrical properties of the GNP nanocomposites (electrical resistivity and conductivity) are influenced by the GNP concentration and follow a percolation law [116,120,131].

The addition of GNPs provides the cementitious matrix with stable repeatable piezoresistive characteristics even after several cyclic quasi-static and dynamic compressive loadings [120]. The electrical resistance change increases with more severe damage and when the measurement is performed closer to the damaged area [132]. The piezoresistive properties of the GNP nanocomposite are strongly affected by the lateral size of the platelets [133]. An increased change in the electrical resistance was recorded when GNPs with larger lateral size (25 μm) were used [133]. GNP with high C/O atomic ratio demonstrate improved electrical conductivity and display piezoresistive characteristics at lower concentrations [130]. A full scale reinforced concrete beam incorporating the GNP nanocomposite was developed by Rehman et al. [117]. Possibly, crack propagation could be successfully predicted using the GNP nanocomposites [117]. A more recent study shows that GNP/concrete can be used to detect damage [134].

#### 2.3.2. Graphene Oxide (GO)

GO consists of a hexagonal carbon network holding hydroxyl and epoxide functional groups on its basal plane and carbonyl and carboxyl groups located at the sheet edges [135]. Typically, the preparation of GO involves three steps, oxidation (in which functional groups containing oxygen were inserted into graphite to make hydrophilic oxide), filtration (in which the remaining ions were removed by using deionized water) and exfoliation (in which GO is subject by ultrasonication) [136]. GO is one of the most generally used 2D nanosheet in cementitious materials [137,138,139]. Two recent reviews provide in depth information on the effect of GO in the cementitious matrix [140,141]. Tanking this into account this review is focused on providing the most recent scientific results that are not included in the previous reviews and also inform on the main effects of GO addition into the cementitious matrix.

GO compared to other types of carbon nanomaterials is easier to disperse in water due to the electrostatic repulsion and hydrophilic nature [142,143]. However, homogeneous distribution in the cementitious matrix is difficult to be achieved due to the presence of alkaline ions, e.g., Ca^2+^, K^+^,Na^+^, OH^–^, in large concentrations, in the fresh state of the matrix [140,141]. The repulsion forces between the GO nanomaterials are depleted resulting to the re-agglomeration of the GO which occurs promptly after their introduction in the cement paste. This hinders the full exploitation of the excellent GO physicochemical and mechanical properties in the cement-based matrix.

Typically, a surfactant in combination with ultrasonication is employed to disperse the GO in the aqueous solution [144,145]. In the majority of the studies a polycarboxylate based superplasticizer, which is fully compatible with the matrix, is proposed as a dispersing agent [146,147,148]. Special attention should be paid on the amount of the dispersant used, and the method that it is used. For example, addition of the dispersing agent at the GO suspension has been found to be much more effective that adding it to the GO-cement mix (GO suspension mixed with cement first) [148]. The molecular structure of the superplasticizer and specifically the length of the side or main chains, the polymer molecular weight and the anchor groups, are some factors that strongly affect their dispersion performance [106].

GO reduces the workability of the cement matrix [149]. GO due to its hydrophilic nature and its large surface area absorbs water. As a result, an increased friction between the cement grains occurs deceasing the flowability of the nanocomposites.

Recent studies have shown that, the addition of GO improves the interfacial transition zone (ITZ) microstructure reducing its thickness and porosity [150,151]. Substantial reduction in water permeability was also reported [152]. Inserting a really small dosage like 0.04–0.05 wt% of GO increases the compressive strength and flexural strength of Portland cement by 33–46% and 59–75%, respectively [152,153]. Similarly, the incorporation of 0.1% reduced GO (rGO) improved the compressive strength along with other properties (water absorption, ultrasonic pulse velocity, carbonation and fire resistance) [154]. Thermal conductivity and thermal diffusivity coefficient were also found to improve when using 1.2 wt % rGO [155].

### 2.4. Nanosilica

**Figure 8:**
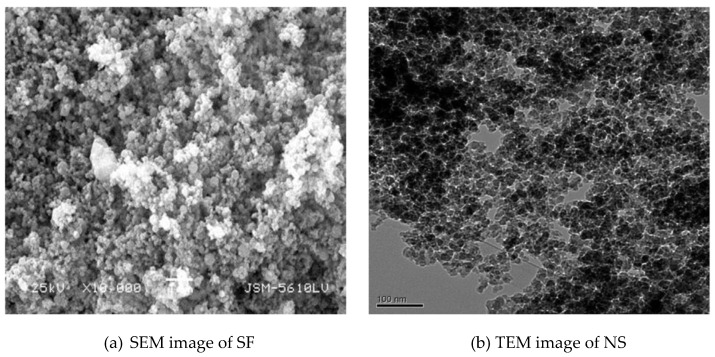
The microstructure of silica fume (SF) and nanosilica (NS). Reproduced from Zhang et al. [3] with permission from Elsevier.

Nanosilica (Nano-SiO_2_, NS) and silica fume (SF), which are categorized as inorganic materials, can significantly improve the pore structure because of the filling effect and positive impact on hydration and microstructure [156]. A recent study reports that hydrothermal SiO_2_ nanoparticles can increase the rate of hydration of clinker minerals by 20–30% [157]. The microstructure of SF and NS is shown in Figure 8.

NS is usually produced by the sol-gel method by the hydrolysis process of trimethylethoxysilate or tetraethoxysilane, and its particle size is often lower than 100 nm. Because of its pozzolanic reactivity and the small particle size (the nanoparticles can fill the spaces between particles of gel of C-S-H, acting as a nano-filler), nanoSiO_2_ can improve the compressive strength of cementitious materials and can make the microstructure denser [158,159,160]. SF and NS are also a new kind of surface protection materials according to a previous study [3]. That is because SF and NS can improve chloride penetration resistance, so can enhance the corrosion resistance ability of concrete. SF may introduce a higher reactivity, that is why it has a greater hydration acceleration effect than NS [161,162].

A previous study [163] showed that the pozzolanic reaction products of nanoSiO_2_ are more compact than those of silica fume. Furthermore, it was concluded that nanoSiO_2_ could make the hardened cement mortar less water-absorbable via exploring either its high pozzolanic reactivity or its filler effect on the surface of the mortar. In another study [164] it was shown that when very small nanoSiO_2_ particles were added in the cementitious materials at a low concentration, they absorbed onto Ca(OH)_2_ hydration product and at the same time acted as nucleation sites, which would advantage the hydration process. Further addition of nanoSiO_2_ up to 5% had negative impact on the microstructure of cementitious composites because of nanoSiO_2_ particles agglomeration. A more recent study, reports that the incorporation of NS refines the pore structure and reduces the pore volume of cement pastes with ultra-high volume fly ash [165]. Similarly, the microstructural studies of Sikora et al. confirmed that NS significantly affects the pore characteristics of concretes, thus resulting in concretes with denser and stronger microstructures [166]. Several researchers have reported that 1% NS is the optimum percentage to enhance the mechanical properties (both the compressive and flexural strength) of the cementitious matrix [167,168,169,170,171]. NS has been also shown to improve the durability of the matrix by reducing its water absorption, capillary absorption, rate of water absorption, co-efficient of water absorption and water permeability compared to normal concrete [158]. It is also reported that the addition of silica nanoparticles reduced the chloride ion penetration significantly [172].

### 2.5. Nano Titanium Dioxide

Nano titanium dioxide (TiO_2_, NT) is a 0D nanomaterial which has unique physical and chemical properties. With NT addition, cementitious composites can become high-performance, multifunctional and environmentally friendly. Generally, NT is in the form of powder, sol and slurry, and its particle is spherical or ellipsoidal [173]. A 45.01% increase in compressive strength [174], a 87.00% increase in flexural strength [175], a 43.48% increase in tensile strength [176], a 27.00% decrease in the shrinkage strain [177], a 43.90% decrease in water vapor penetration coefficient [178], a 60.87% increase in chloride ion penetration resistance [179], a 75.03% decrease in the corrosion rate in NaCl solution [174], a 49.81% decrease in the corrosion rate in H_2_SO_4_ solution [174] and a 59.11% decrease in the coefficient of water absorption [180] has been reported with the incorporation of NT in cementitious matrices. In addition, NT-engineered cementitious composites have an average NOx abatement of 45% [181] and an organic degradation efficiency of 78% [182].

**Figure 9:**
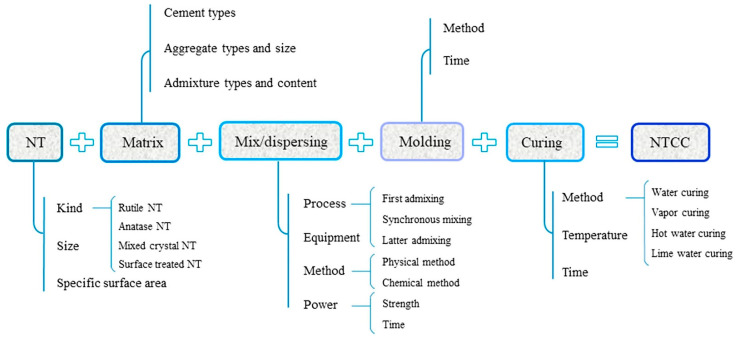
Schematic presentation of nano titanium dioxide/cementitious composites (NTCC) production methods. Reproduced from Li et al. [173] with permission from Elsevier.

The different methods used to produce nano titanium dioxide/cementitious composites are shown schematically in Figure 9. It is observed that researchers should take various decisions, from the specific characteristics of the NT used to the curing method followed to prepare the nanocomposites. Most of the studies state that the most crucial issues considering nanoTiO_2_ successful implementation are the NT concentration, dispersion method and mix proportions of the composites [173].

NT-cementitious composites can be used as [173]: (i) Pavement materials; (ii) Exterior wall materials, harmful gas and bacteria could be adsorbed on the nano titanium dioxide surface and neutralized via UV light provided by sun [183]; (iii) Surface materials [184]; and (iv) Inner wall materials, it can get harmful gas off such as formaldehyde, benzene and increase the indoor air quality [185]. Finally, it is important to report that NT can become environmental contaminants upon any accidental leakage, and extra research is a necessity to investigate its impact on human health [173].

## 3. Conclusions

Nanoscale materials have recently gained excessive attention due to their excellent physicochemical characteristics such as improved mechanical, electrical and thermal properties, low density and excellent thermal stability [186,187,188,189,190,191,192,193,194]. The present article targets to overview the recent studies published on the use of nanomaterials in cementitious composites. Cementitious composites have many drawbacks such as low tensile capacity, poor deformation performance, high cracking tendency, high porosity and the production of cement for concrete is contributing nearly 8% of global carbon dioxide emission. With the addition of nanomaterials in cementitious composites it is possible to overcome these issues and develop a new generation of high-performance, and multifunctional cementitious composites.

The most widely used nanomaterials in cementitious composites are the carbon nanotubes followed by graphene-based materials, nanosilica and nano-TiO_2_. The main factors affecting the nanocomposites performance are the nanomaterials’ dosage and dispersion state. Carbon nanotubes can be used to improve the compressive strength, flexural strength, fracture toughness, hydration, freeze-thaw resistance and electromagnetic interference of the cementitious matrix as well as decrease its porosity. The use of graphene has a higher impact on the tensile strength and Young’s modulus of the matrix compared to the other nanomaterial types. Nanosilica can be used to significantly improve the microstructure of cementitious nanocomposites.

Nanotechnology application has the potential to make breakthroughs in concrete technology. In order to achieve that, future research should be application specific. That is focusing on developing nanocomposites with targeted improved properties intended for specific applications. In this aspect, a relationship should be established between optimum quantity and characteristics of the nanomaterials. Additionally, more research is needed on the performance of concrete with nanomaterials as researchers have mainly focused on cement paste and mortar. Finally, more research is needed on the development of theoretical models that can predict the performance of the cement-based nanocomposites as function of the nanomaterials’ concentration.

## Data Availability

The data presented in this study are available upon request from the corresponding authors.
